# Supplementary material for: The δ subunit and NTPase HelD institute a two-pronged mechanism for RNA polymerase recycling
Source: Nat Commun. 2020 Dec 18;11:6418. doi: 10.1038/s41467-020-20159-3 (PMC7749165; doi:10.1038/s41467-020-20159-3)
Supplement: Supplementary file 1 — Supplementary Information [file 41467_2020_20159_MOESM1_ESM.pdf]

Supplementary Information

The δ subunit and NTPase HelD institute a two-pronged mechanism for RNA polymerase recycling

Hao-Hong Pei[1], Tarek Hilal[2], Zhuo A. Chen[3], Yong-Heng Huang[1], Yuan Gao[1], Nelly Said[1], Bernhard Loll[1], Juri Rappsilber[3,4], Georgiy A. Belogurov[5], Irina Artsimovitch[6], Markus C. Wahl[1,7,*]

[1] Freie Universität Berlin, Institute of Chemistry and Biochemistry, Laboratory of Structural Biochemistry, Takustraβe 6, D-14195 Berlin, Germany

[2] Freie Universität Berlin, Institute of Chemistry and Biochemistry, Research Center of Electron Microscopy and Core Facility BioSupraMol, Fabeckstr. 36a, 14195 Berlin, Germany

[3] Technische Universität Berlin, Institute of Biotechnology, Bioanalytics, Gustav-Meyer-Allee 25, 13355 Berlin Berlin, Germany

[4] University of Edinburgh, Wellcome Centre for Cell Biology, Edinburgh EH9 3BF, UK

[5] University of Turku, Department of Biochemistry, FIN-20014 Turku, Finland

[6] The Ohio State University, Department of Microbiology and Center for RNA Biology, Columbus, OH, USA

[7] Helmholtz-Zentrum Berlin für Materialien und Energie, Macromolecular Crystallography, Albert-Einstein-Straße 15, D-12489 Berlin, Germany

* Correspondence to: markus.wahl@fu-berlin.de

Supplementary Tables

Supplementary Table 1: Nucleic acids and bacterial strains.

**PCR primers**	
helD	
Forward	5’-TGCGCGGATCCATGAATCAGCAGGATAAGGAATGGAAGG-3’
Reverse	5’-TACCGCTCGAGTCATTCAGCAATCTGATATAAGTGAGG-3’
rpoE	
Forward	5’-GTCGGCCATGGGTATCAAACAATATTCACAGG-3’
Reverse	5’-GAGCCAAGCTTATTTAATTTCCTCTTCTTCATCATC-3’
rpoE-NTD	
Forward	5’-CCCATGGGGATGGGTATCAAACAATATTCACAG-3’
Reverse	5’-CCCTCGAGTTCTTCATCAAGCTGATCATAAG-3’
sigA	
Forward	5’-CCCATGGGGATGGCTGATAAACAAACCC-3’
Reverse	5’-CCAAGCTTTTATTCAAGGAAATCTTTCAAACG-3’
**Scaffold**	
ntDNA	5’-GCCGAGCAGCGTAGCATTACTTGTGAGCGGATAAC-3’
tDNA[a]	5’-GTTATCCGCTCACAATGCCACACGCGCTGCTCGGC-3’
RNA 9-mer	5’-CGUGUGGCA-3’
B. subtilis **strains**	
MH5636 (*rpoC-His*$_{10}$)	Reference [1]
LK782 (*rpoC-His*$_{10}$, *helD::MLS*)	Reference [2]
LK1032 (*rpoC-His*$_{10}$, *rpoE::kan*, *helD::MLS*)	Reference [2]

[a] Region complementary to RNA 9-mer in red

Supplementary Table 2: CryoEM data collection and refinement statistics.

Dataset	Monomeric RNAP-δ-HelD	Dimeric RNAP-δ-HelD
PDB ID	6ZCA	6ZFB
EMDB ID	EMD-11104	EMD-11105
**Data collection**		
Pixel size (Å/px)	0.832	
Defocus range (µm)	0.5 – 2.5	
Voltage (kV)	300	
Electron dose (e^-/Å^2)	40	
Number of frames	29	
Micrographs	9123	
Particle images	81279	176374
**Refinement**		
Global resolution $FCS_{0.143}$ (Å)	4.23	3.85
Local resolution range (Å)	2.6 – 7.0	2.8 – 6.5
CC mask	0.76	0.78
CC volume	0.75	0.77
Model composition		
Non-hydrogen atoms	28,724	57,521
Protein residues	3,640	7,313
Zn^{2+}/Mg^{2+} ions	1	2
Rmsd from ideal geometry		
Bond lengths (Å)	0.003	0.002
Bond angles (°)	0.535	0.559
Ramachandran plot (%)		
Favored	92.3	91.4
Allowed	7.7	8.4
Outliers	0.0	0.2
Model quality[a]		
Clash score	13.4	8.2
Rotamer outliers	0.0	4.0
Overall score	2.1	2.6

[a] Assessed using MolProbity[3].

Supplementary Table 3: Regions of RNAP and factors discussed in the text.

	E. coli	*B. subtilis*
**β**		
β1-lobe (protrusion)	31-139/456-512	33-128/412-468
β2-lobe	151-444	156-400
β SI1	226-350	281-367
β gate loop	359-388	229-258
β fork loop	533-599	489-557
β protrusion	450-507	406-463
β connector	814-839/1048-1065	773-798/907-924
β flap	830-1058	789-917
β flap tip	887-915	846-874
β flap tip arms	890-899/910-914	849-855/866-873
β SI2	938-1040	-
C-terminal β clamp	1233-1342	1038-1113
β switch 3	1247-1268	1052-1073 (not resolved)
**β‘**		
β' ZBD	35-107	28-97
β' zipper	36-61	26-51
β' clamp	16-342/1318-1344	6-332/1132-1158
N-terminal β‘clamp	132-190	122-176
β' lid	250-264	239-253
β' clamp helices	265-307	254-296
β' rudder	308-327	298-316
β' switch 1	1326-1327	1139-1140
β' switch 2	330-349	319-337 (not resolved)
β' dock	369-420	358-409
β' shelf	787-931	791-927
β' SI3	943-1130	-
β' jaw	1135-1317	952-1131
β' C-term	1318-1375	1132-1159
2° channel	480-790	473-794
β' bridge helix	768-850	777-853 (break 780-787)
Trigger loop	915-941/1130-1148	938-952
**δ**		
NTD	-	1-90
CTR	-	91-173
**HelD**		
NTD	-	1-200
D1a/D1b	-	201-338/491-603
Ins	-	339-490
D2	-	604-774

Supplementary Table 4: HelD and δ inter-molecular crosslinks in RNAP$^{\Delta\delta\Delta HelD}$-δ-HelD.[a]

Protein/region 1	Residue 1	Protein/region 2	Residue 2	Highest score	Cα-Cα (Å)
HelD	442	β' rudder	312	13.34	13.7
HelD	37	β' trigger loop	948	16.376	12.2
HelD	37	β' trigger loop	949	15.601	9.3
HelD	37	β' trigger loop	939	14.106	19.6
HelD	37	β' bridge helix	831	8.929	13.3
HelD	37	β' bridge helix	832	10.791	13.9
HelD	37	β' bridge helix	834	13.462	11.2
HelD	37	β' bridge helix	837	9.694	15.4
HelD	433	β' bridge helix	800	12.841	14.9
HelD	437	β' bridge helix	793	14.772	17.1
HelD	45	β' 2° channel	617	10.918	14.2
HelD	443	β' clamp	314	11.393	7.9
HelD	443	β' clamp	317	7.78	8.5
HelD	392	β 2-lobe	177	11.621	16.9
HelD	393	β 2-lobe	177	14.001	14.4
HelD	397	β 2-lobe	190	11.908	15.4
HelD	386	β 2-lobe	223	16.669	9.3
HelD	388	β 2-lobe	223	9.172	11.2
HelD	392	β 2-lobe	223	15.132	12.6
HelD	392	β 2-lobe	223	15.132	12.6
HelD	393	β 2-lobe	223	15.204	11.2
HelD	396	β 2-lobe	223	11.95	15.8
HelD	397	β 2-lobe	223	14.267	15.8
HelD	399	β 2-lobe	223	13.703	20.4
HelD	537	β 2-lobe	216	11.78	13.6
HelD	535	β 2-lobe	223	11.126	14.7
HelD	536	β 2-lobe	223	13.548	13.1
HelD	97	β' jaw	983	13.815	16.9
HelD	97	β' jaw	1006	12.288	15.5
HelD	97	β' jaw	1007	10.609	14.8
HelD	97	β' jaw	1010	11.021	12.7
HelD	93	β' jaw	1011	8.759	12.4
HelD	175	β' jaw	1012	11.727	15.5
HelD	425	β' jaw	1031	13.164	23.5
HelD	424	β' jaw	1032	11.964	21.0
HelD	426	β' jaw	1032	16.241	18.9
HelD	427	β' jaw	1032	18.178	21.4
HelD	425	β' jaw	1033	10.684	24.6
δ	83	β' jaw	1032	12.628	19.3
δ	85	β' jaw	1032	15.907	16.6
δ	87	β' jaw	1032	11.341	16.0
δ	90	β' jaw	1032	13.574	19.4

[a] Color intensity scales with proximity of crosslinked RNAP elements to the active site.

Supplementary Figures

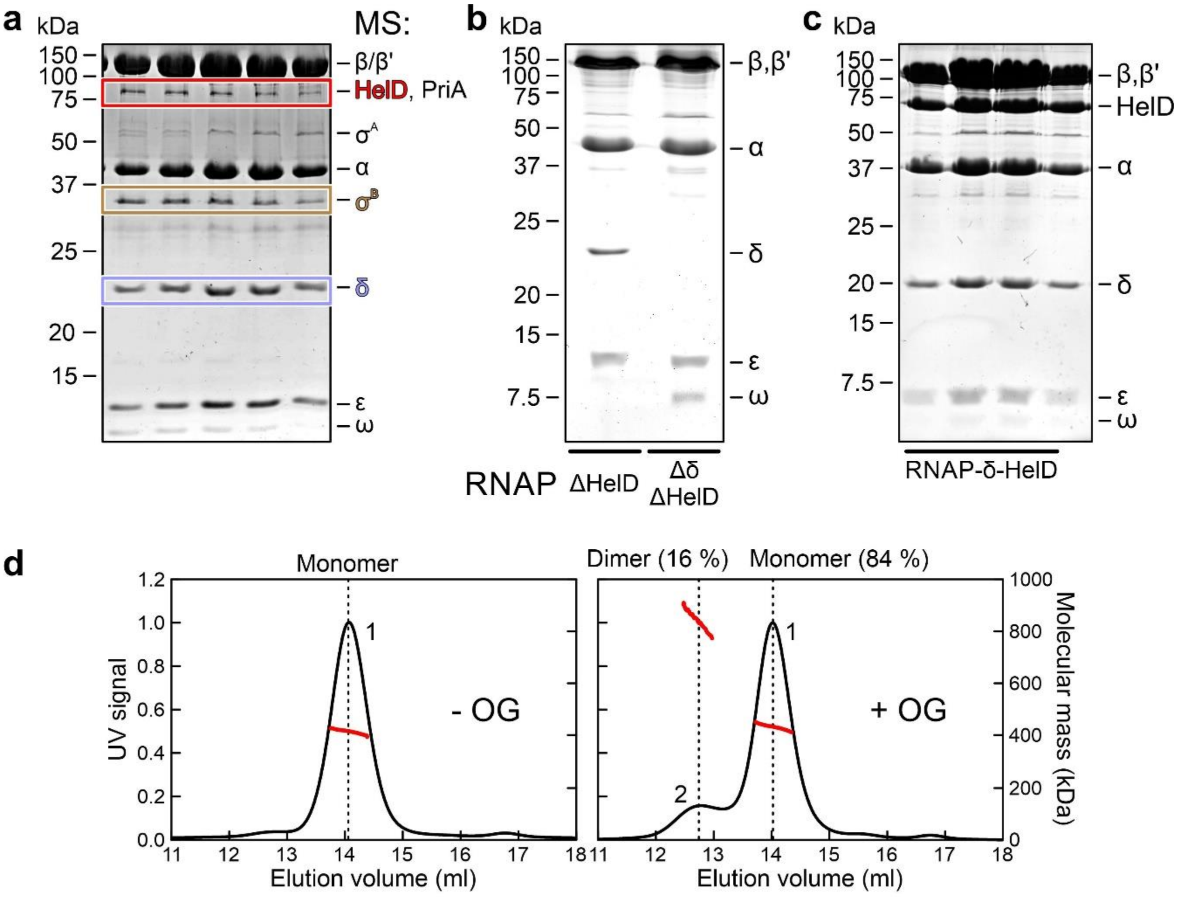

M [kDa]	- OG	+ OG	Theor.
Peak 1 / Mon.	415	433	454.7
Peak 2 / Dimer	-	836	909.4

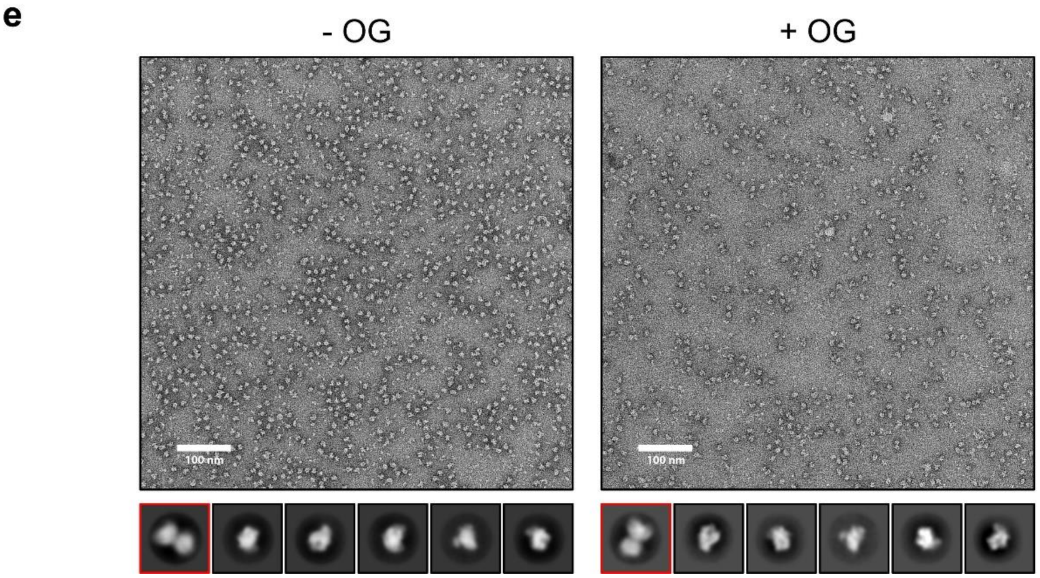

Supplementary Figure 1: Complex preparations.

a, Peak fractions of a SEC run of *B. subtilis* RNAP, affinity-enriched from stationary phase cells, monitored by SDS-PAGE. MS, proteins were identified by cutting out the respective bands, in-gel trypsin digestion and MS-based fingerprinting. PriA, primosomal replication factor Y.

b, **c**, SDS-PAGE analysis of purified RNAP$^{\Delta HelD}$ and RNAP$^{\Delta\delta\Delta HelD}$ (**b**) and of a SEC run of RNAP-δ-HelD (**c**). Experiments shown in (a-c) were repeated independently at least three times with similar results.

d, SEC/multi-angle light scattering analysis of RNAP-δ-HelD used for cryoEM analysis in buffer lacking (-OG) or containing (+OG) 0.15 % (w/v) n-octylglucoside (critical micellar concentration 0.6 % [w/v]). Black traces, UV signals; red lines, molecular mass estimates across the peaks. Molecular masses deduced are listed in the bottom table compared to the theoretical (theor.) molecular masses for RNAP-δ-HelD (Mon.) and (RNAP-δ-HelD)$_2$ (Dimer). About 16 % of the sample traverses the column as intact (RNAP-δ-HelD)$_2$ dimers in the presence of n-octylglucoside.

e, Top, negative stain EM micrographs of RNAP-δ-HelD in buffer lacking (-OG) or containing (+OG) 0.15 % (w/v) n-octylglucoside. Scale bars, 100 nm. Bottom, 2D class averages of picked particle images. Classes boxed red unequivocally indicate the presence of (RNAP-δ-HelD)$_2$ dimers in both samples. For negative stain EM analyses, 25 high-quality micrographs were recorded from one biological sample.

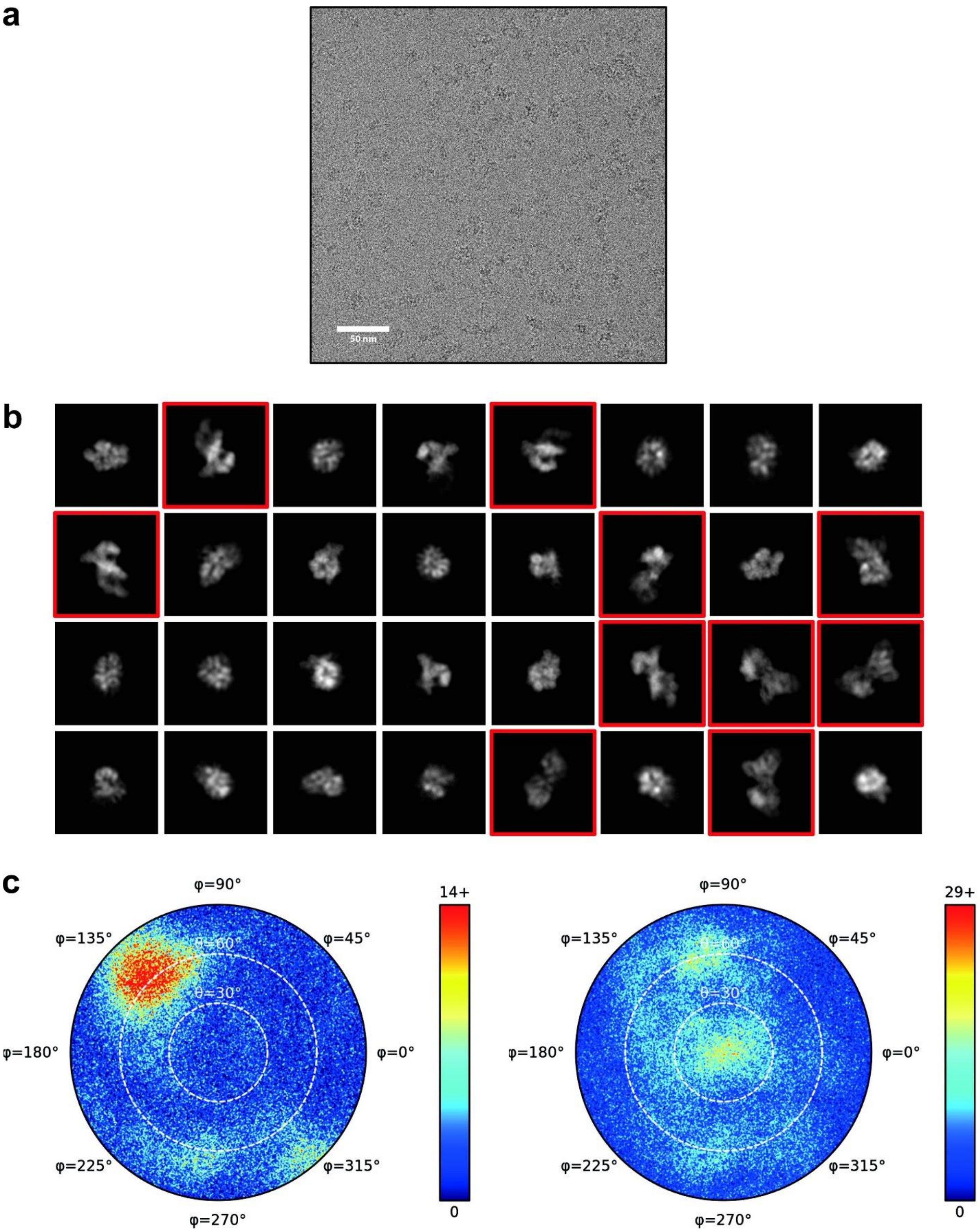
a
50 nm
b
c
φ=90°
φ=135°
φ=45°
θ=60°
θ=30°
φ=180°
φ=0°
φ=225°
φ=315°
φ=270°
14+
0
φ=90°
φ=135°
φ=45°
θ=60°
θ=30°
φ=180°
φ=0°
φ=225°
φ=315°
φ=270°
29+
0

Supplementary Figure 2: CryoEM data.

a, Representative cryo electron micrograph of RNAP-δ-HelD particles. Scale bar, 50 nm.

b, 2D class averages of RNAP-δ-HelD particles. Red boxes indicate classes representing RNAP-δ-HelD dimers.

c, Polar plots of particle orientations in the monomeric (left) and dimeric (right) RNAP-δ-HelD datasets. Legends, color codes for particle numbers.

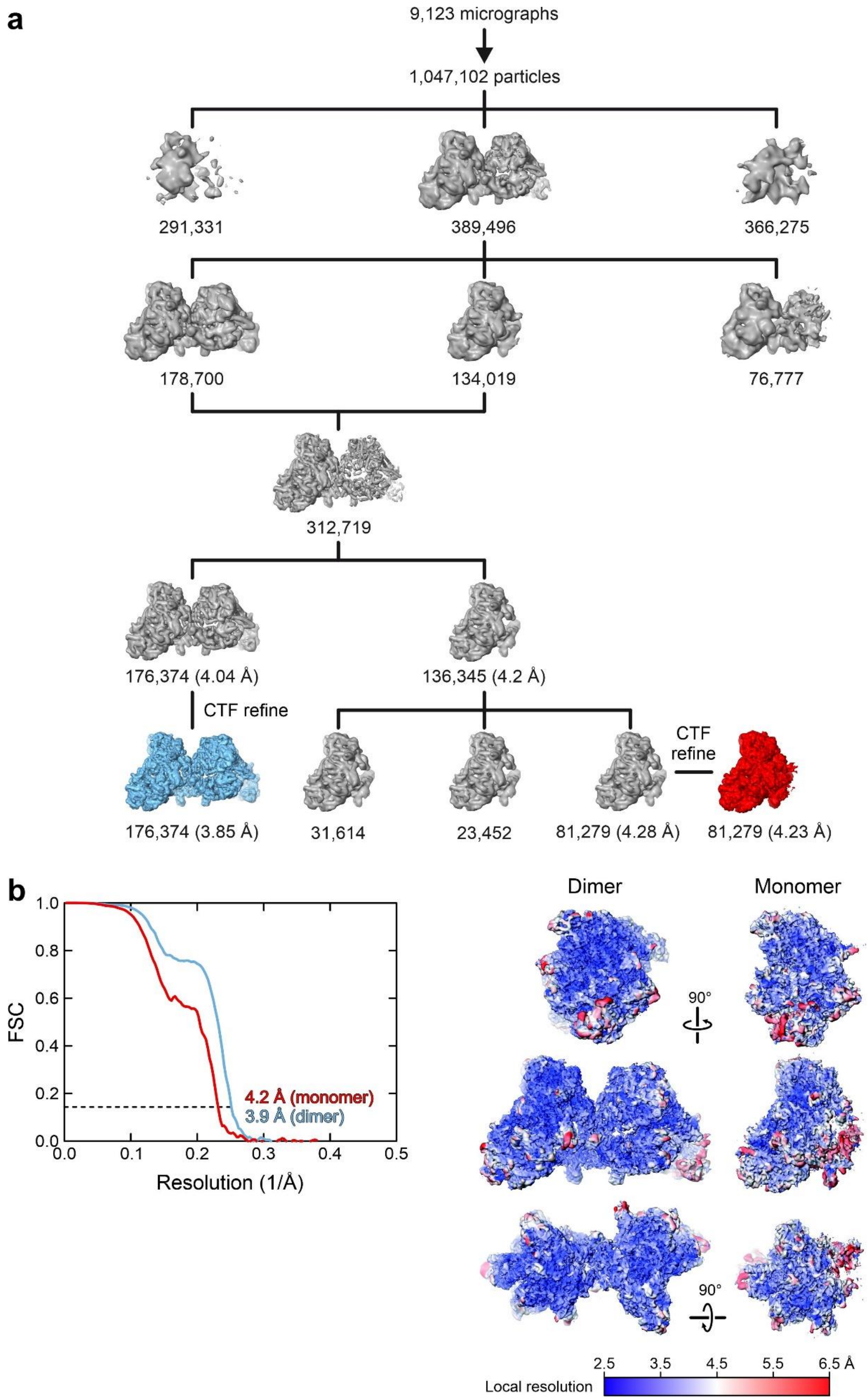

a
9,123 micrographs
1,047,102 particles
291,331
389,496
366,275
178,700
134,019
76,777
312,719
176,374 (4.04 Å)
136,345 (4.2 Å)
CTF refine
CTF refine
176,374 (3.85 Å)
31,614
23,452
81,279 (4.28 Å)
81,279 (4.23 Å)
b
FSC
Resolution (1/Å)
4.2 Å (monomer)
3.9 Å (dimer)
Dimer
Monomer
90°
90°
Local resolution
2.5
3.5
4.5
5.5
6.5 Å

73

Supplementary Figure 3: Hierarchical clustering analysis.

a, Hierarchical clustering analysis.

b, Left, Fourier shell correlation (FSC), indicating nominal resolutions of 4.2 Å and 3.9 Å for the overall cryoEM maps of monomeric and dimeric RNAP-δ-HelD complexes, respectively, according to the $FSC_{0.143}$ criterion. Right, orthogonal views of the cryoEM map for dimeric RNAP-δ-HelD, colored according to the local resolution in different regions. Legend, color code for local resolution.

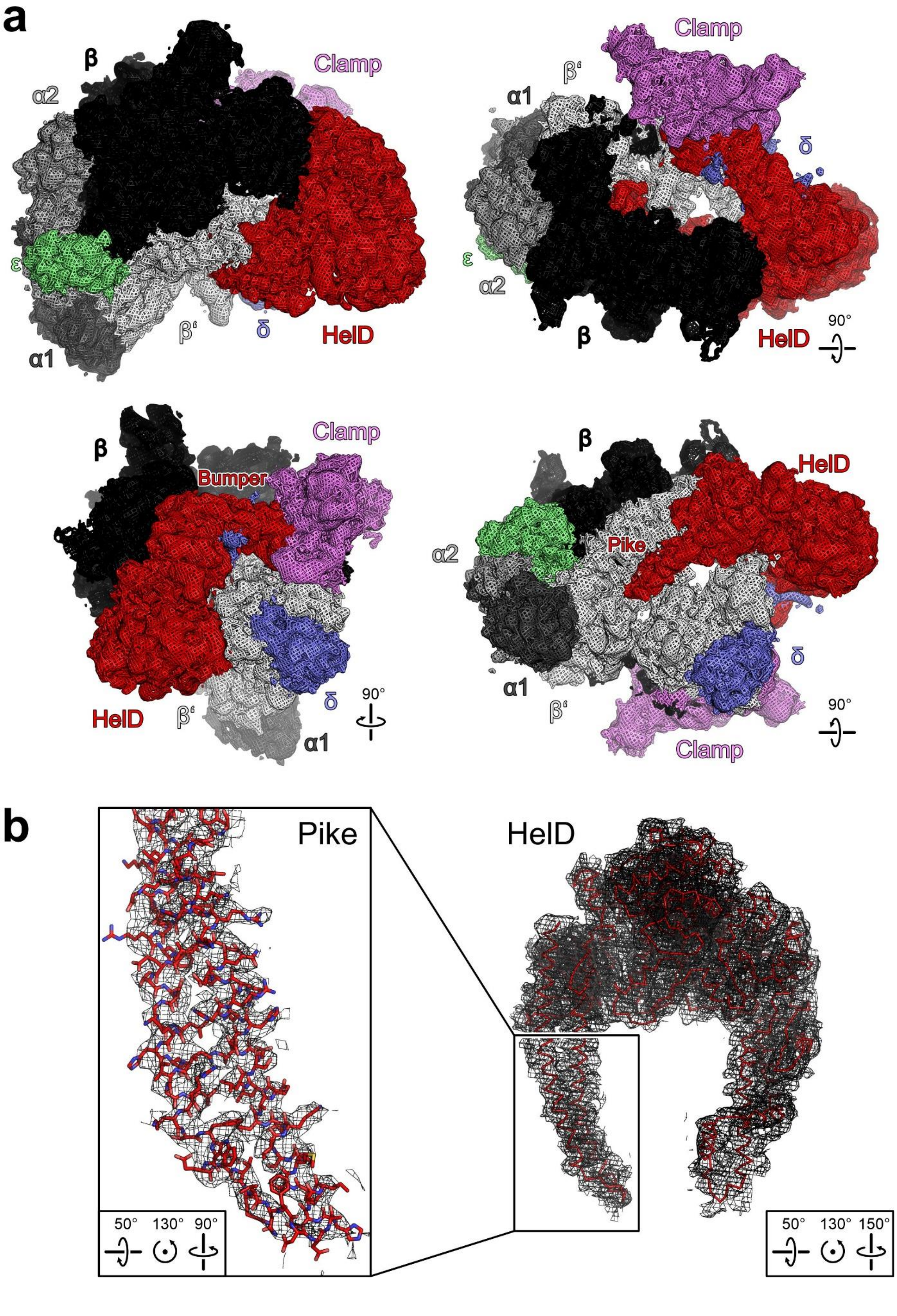
a
β
Clamp
α2
ε
β'
δ
HelD
α1
Clamp
β'
α1
δ
ε
α2
β
HelD
90°
β
Clamp
Bumper
HelD
β'
δ
α1
90°
β
HelD
α2
Pike
δ
α1
β'
Clamp
90°
b
Pike
HelD
50° 130° 90°
50° 130° 150°

Supplementary Figure 4: CryoEM map.

a, Orthogonal views of cryoEM density of the monomeric RNAP-δ-HelD complex contoured at the 7 σ level.

b, CryoEM density covering HelD in the monomeric RNAP-δ-HelD complex contoured at the 7 σ level. HelD is shown as a red ribbon. Inset, close-up view of the cryoEM density around $HelD^{Pike}$. $HelD^{Pike}$ is shown as sticks; carbon, red; nitrogen, blue; oxygen light red; sulfur, yellow. Rotation symbols indicate views relative to panel (**a**), upper left.

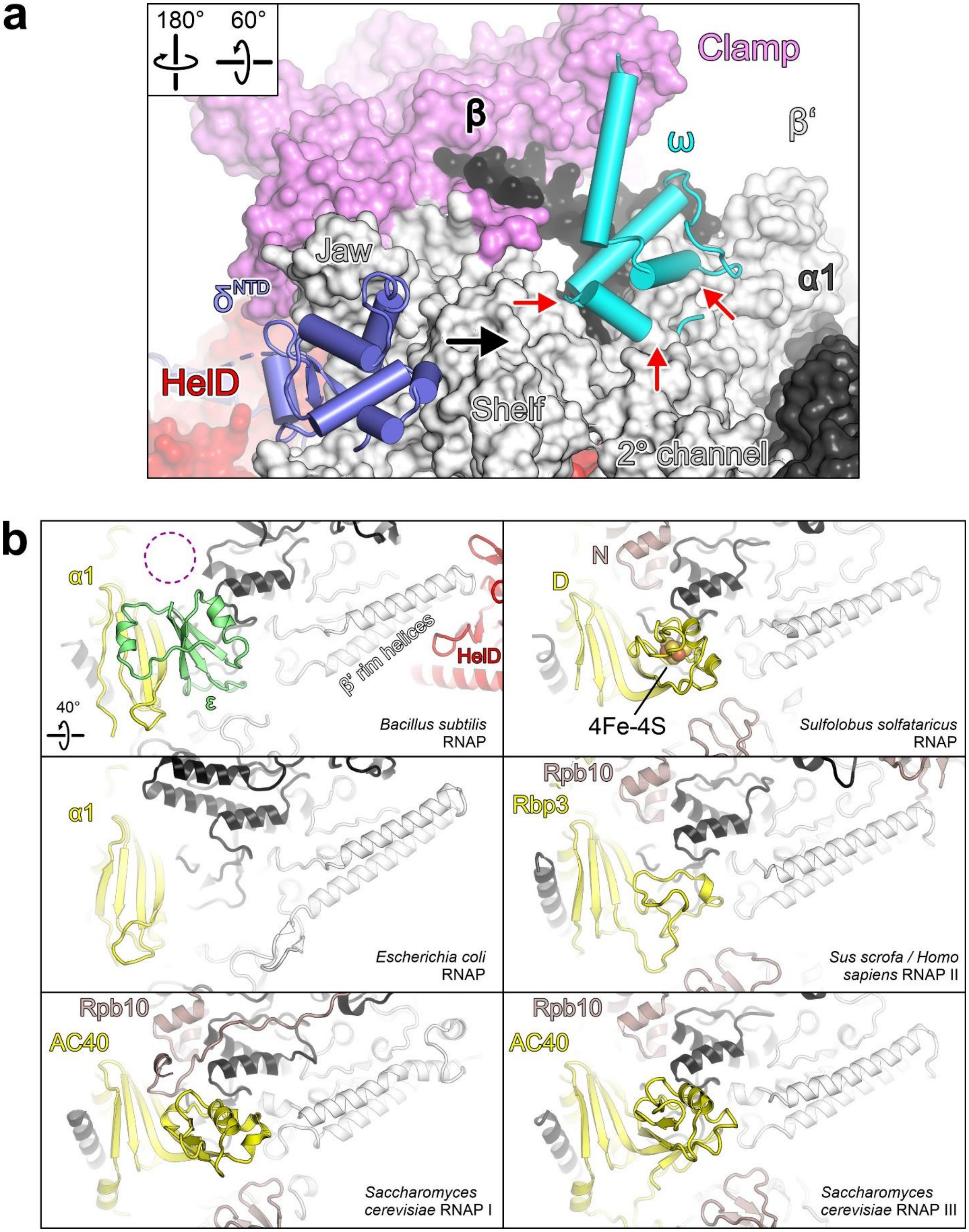
a
180°
60°
Clamp
β
β'
ω
Jaw
δ^NTD
α1
HelD
Shelf
2° channel
b
α1
ε
β' rim helices
HelD
40°
Bacillus subtilis
RNAP
N
D
4Fe-4S
Sulfolobus solfataricus
RNAP
α1
Escherichia coli
RNAP
Rpb10
Rbp3
Sus scrofa / Homo
sapiens RNAP II
Rpb10
AC40
Saccharomyces
cerevisiae RNAP I
Rpb10
AC40
Saccharomyces
cerevisiae RNAP III

Supplementary Figure 5: HelD-δ/ω competition and ε subunit.

a, Close-up view on δ^{NTD} and ω binding regions with ω transferred from an *E. coli* EC (PDB ID 6ALH) by superpositioning of the β subunits. δ^{NTD} and HelD displace the β' shelf and other secondary channel elements (black arrow), leading to steric hindrance of ω binding (red arrows). **b**, Archaeal and eukaryotic nuclear RNAPs contain homologs of the bacterial α1 subunit (D, Rpb3 and AC40 of archaeal RNAP, eukaryotic RNAP II and eukaryotic RNAP I/III, respectively), which comprise small domains that occupy analogous positions as the ε subunit in *B. subtilis* RNAP. In some archaeal and eukaryotic RNAPs, these small domains bind an 4Fe-4S cluster.[4] α1, α2, β, β' subunits and their homologs are colored yellow, gray, black and white, respectively; ε subunit, lime; HelD, red; subunits specific to archaeal and eukaryotic RNAPs, dark salmon. Dashed circle, cavity in *B. subtilis* RNAP, but not in the *E. coli* enzyme, that could accommodate an equivalent of the archaeal subunit N (Rpb10 in eukaryotic RNAP I, II and III), but remains unoccupied in the present structures. The illustration was prepared using structures with PDB IDs 3HKZ, 6ALH, 6GMH, 4C2M and 6TUT.

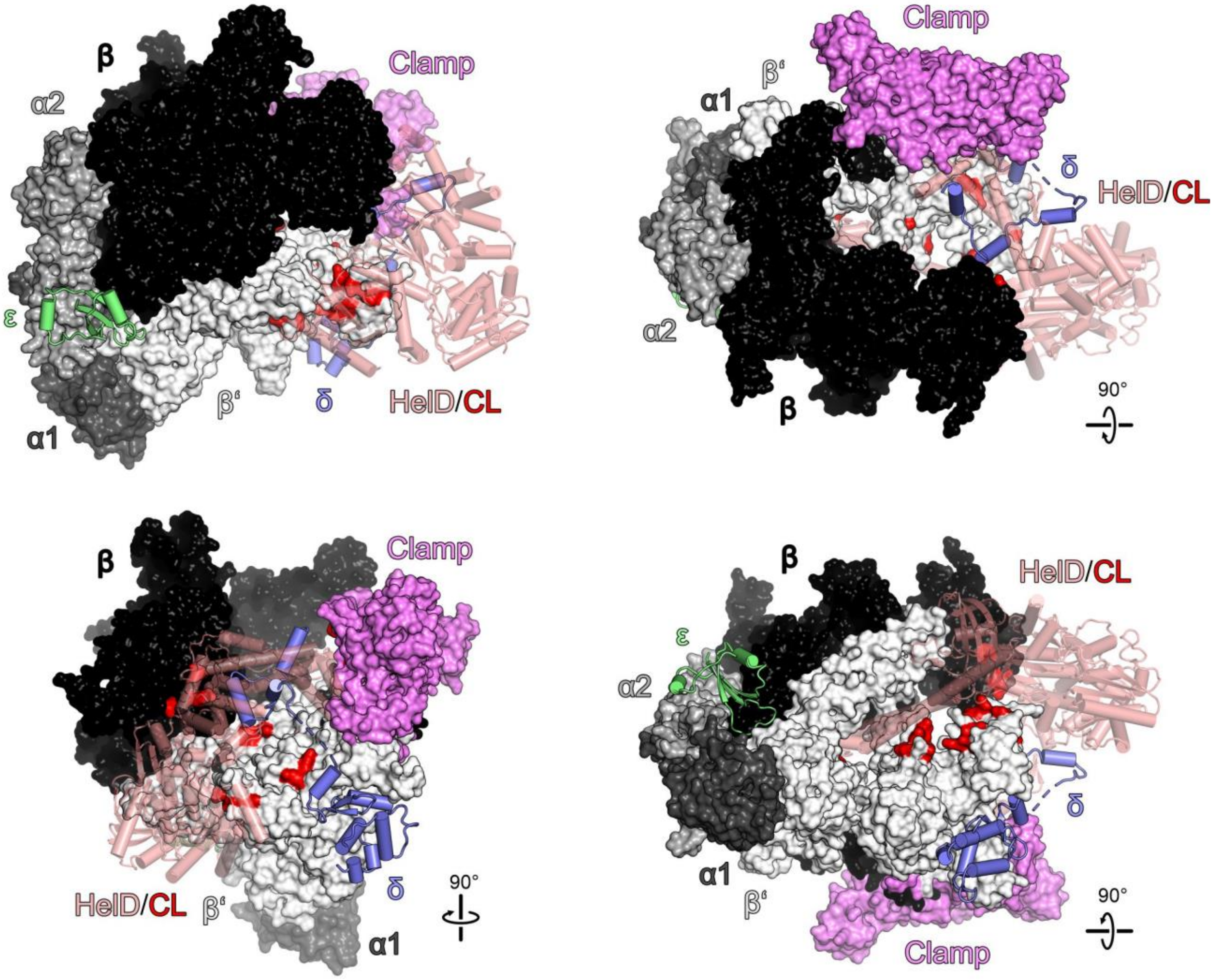

Supplementary Figure 6: Mapping of CLs to the structure.

Mapping of HelD crosslinks to the surface of RNAP. HelD, semi-transparent, light red; crosslinked RNAP residues (CL), red.

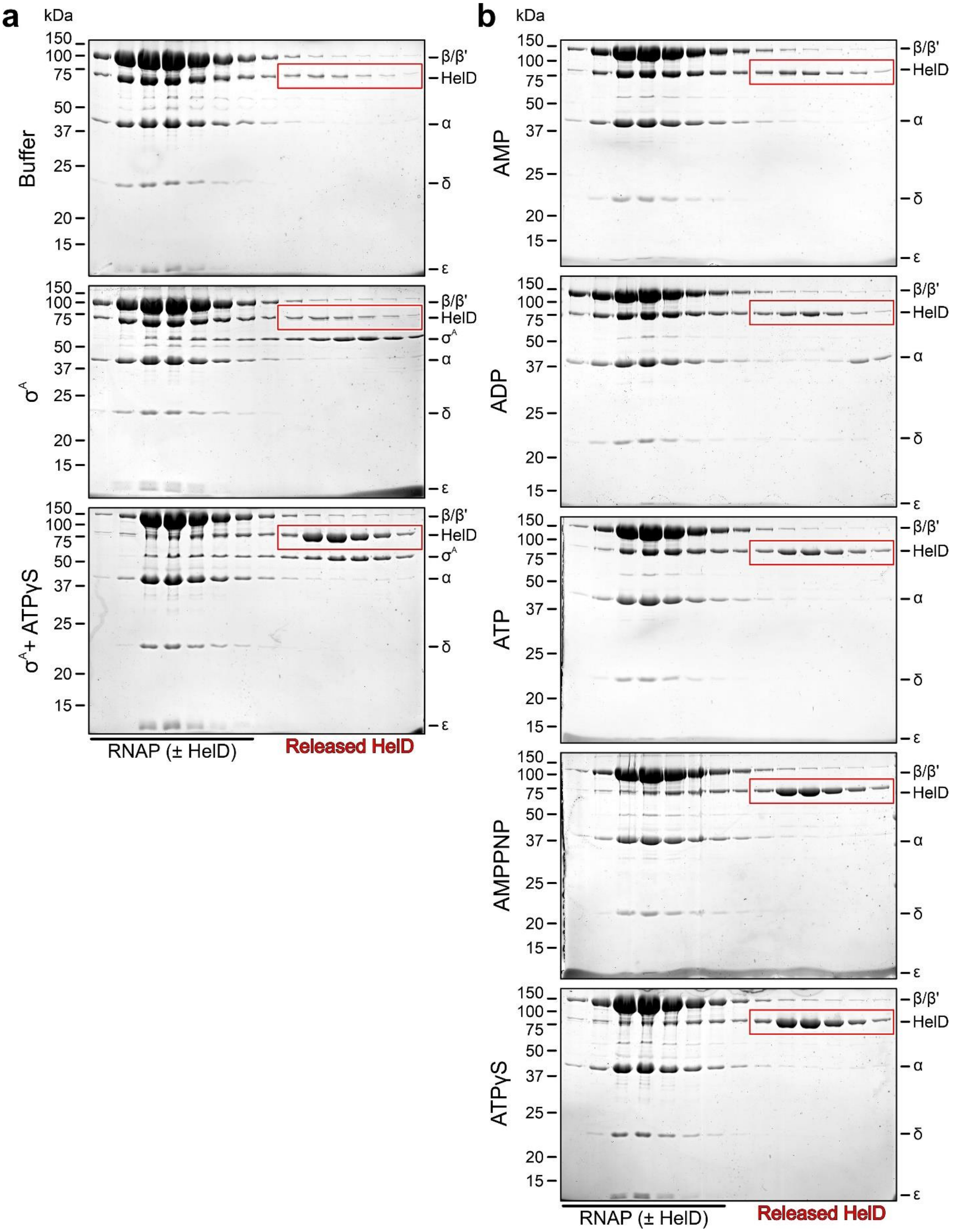
a
kDa
150
100
75
50
37
25
20
15
β/β'
HelD
α
δ
ε
Buffer
σ^A
σ^A+ ATPγS
RNAP (± HelD)
Released HelD
b
AMP
ADP
ATP
AMPPNP
ATPγS

Supplementary Figure 7: HelD release.

a, SDS-PAGE analysis of SEC runs after treating RNAP-δ-HelD with buffer, σ^A or σ^A/ATPγS, as indicated on the left.

b, SDS PAGE gels of which the β/β'/HelD sections are shown in Fig. 7b. Experiments shown in (a) and (b) were repeated independently at least twice with similar results.

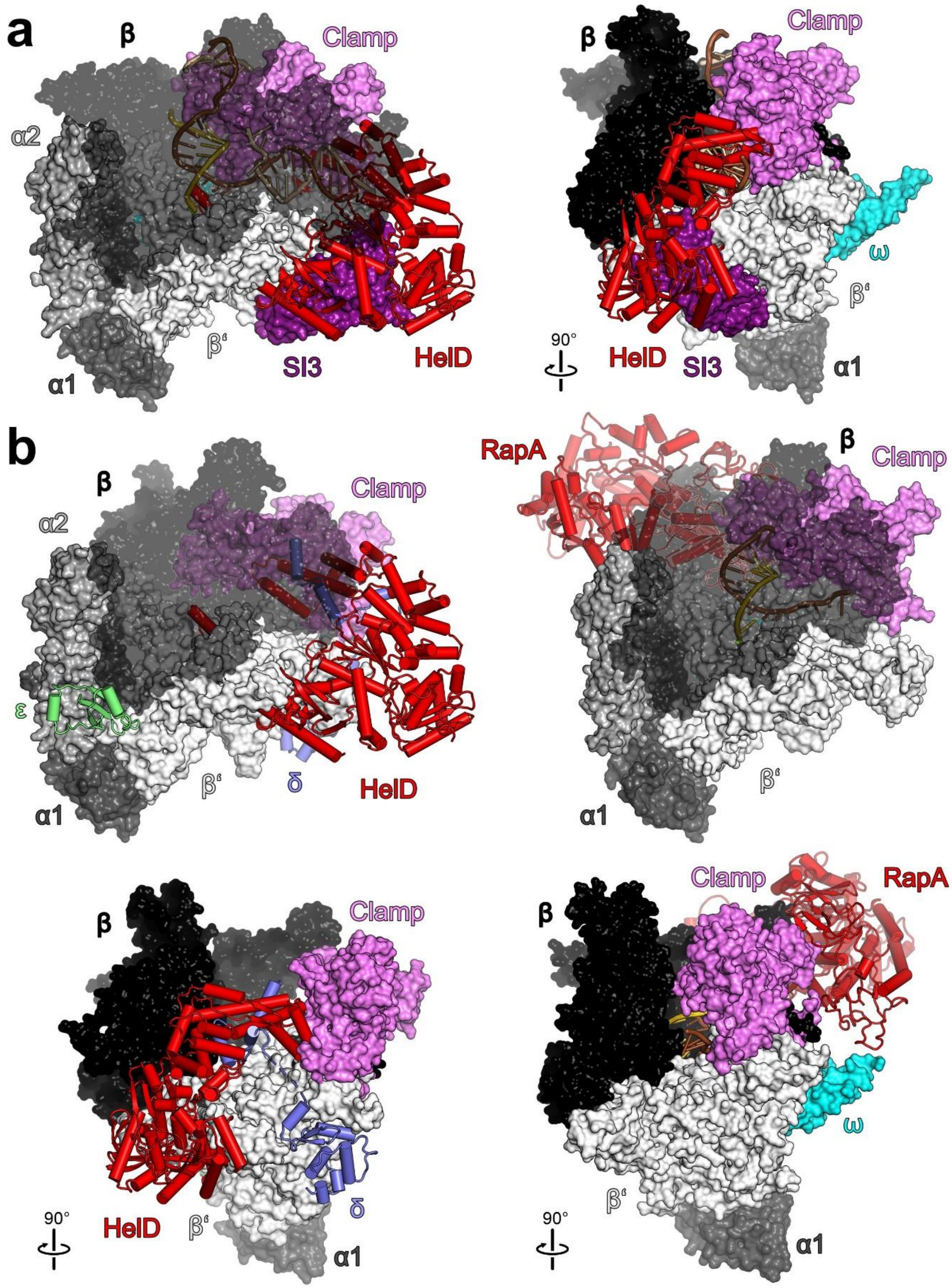
a
β
Clamp
α2
β'
SI3
HelD
α1
β
Clamp
ω
β'
90°
HelD
SI3
α1
b
β
Clamp
α2
ε
β'
δ
HelD
α1
RapA
β
Clamp
β'
α1
β
Clamp
90°
HelD
β'
δ
α1
β
Clamp
RapA
ω
β'
90°
α1

Supplementary Figure 8: Comparison to SI3 RNAPs and a RapA complex.

a, Orthogonal views of HelD transferred on an *E. coli* EC (PDB ID 6ALH) by superpositioning of the β subunits, showing that HelD would clash with SI3 (purple).

b, Comparison of the RNAP-δ-HelD complex (left) to an *E. coli* RapA-modified EC (right; PDB ID 4S20), illustrating how HelD and RapA attack the enzyme from opposite flanks (top), and that RapA binds RNAP in an EC-like closed conformation (bottom).

Supplementary References

1. Qi, Y. & Hulett, F.M. PhoP-P and RNA polymerase sigmaA holoenzyme are sufficient for transcription of Pho regulon promoters in Bacillus subtilis: PhoP-P activator sites within the coding region stimulate transcription in vitro. *Mol. Microbiol.* **28**, 1187-1197 (1998).
2. Wiedermannova, J. et al. Characterization of HelD, an interacting partner of RNA polymerase from Bacillus subtilis. *Nucleic Acids Res.* **42**, 5151-5163 (2014).
3. Williams, C.J. et al. MolProbity: More and better reference data for improved all-atom structure validation. *Protein Sci.* **27**, 293-315 (2018).
4. Hirata, A., Klein, B.J. & Murakami, K.S. The X-ray crystal structure of RNA polymerase from Archaea. *Nature* **451**, 851-854 (2008).
