# Supplementary material for: The δ subunit and NTPase HelD institute a two-pronged mechanism for RNA polymerase recycling
Source: Nat Commun. 2020 Dec 18;11:6418. doi: 10.1038/s41467-020-20159-3 (PMC7749165; doi:10.1038/s41467-020-20159-3)
Supplement: Supplementary file 3 — Description of Additional Supplementary Files [file 41467_2020_20159_MOESM3_ESM.pdf]

Description of Additional Supplementary Files

Supplementary Data 1: CLMS results.

Description: Inter-molecular crosslinks in $RNAP^{\Delta\delta\Delta HelD}$, $RNAP^{\Delta\delta\Delta HelD}$-δ, $RNAP^{\Delta\delta\Delta HelD}$-HelD and $RNAP^{\Delta\delta\Delta HelD}$-δ-HelD.
